# Early Prediction of Abdominal Aortic Aneurysm Rupture Risk Using Numerical Biomechanical Analysis

**DOI:** 10.3390/diagnostics15010025

**Published:** 2024-12-26

**Authors:** Kristina Grassl, Thomas C. Gasser, Florian K. Enzmann, Alexandra Gratl, Josef Klocker, David Wippel, David C. Walcher, Elke R. Gizewski, Sabine H. Wipper

**Affiliations:** 1 Department of Vascular Surgery, Medical University of Innsbruck, 6020 Innsbruck, Austria; florian.enzmann@i-med.ac.at (F.K.E.); alexandra.gratl@i-med.ac.at (A.G.); josef.klocker@i-med.ac.at (J.K.); david.walcher@student.i-med.ac.at (D.C.W.);; 2 KTH Solid Mechanics, Department of Engineering Mechanics, School of Engineering Sciences, KTH Royal Institute of Technology, 171 77 Stockholm, Sweden; gasser@kth.se; 3 Department of Radiology, Medical University of Innsbruck, 6020 Innsbruck, Austria; radiologie@i-med.ac.at

**Keywords:** rupture risk, biomechanical analysis, abdominal aortic aneurysm, peak wall stress, finite element analysis

## Abstract

**Objective**: We aimed to predict patient-specific rupture risks and growth behaviors in abdominal aortic aneurysm (AAA) patients using biomechanical evaluation with finite element analysis to establish an additional AAA repair threshold besides diameter and sex. **Methods**: A total of 1219 patients treated between 2005 and 2024 (conservative and repaired AAAs) were screened for a pseudo-prospective single-center study. A total of 15 ruptured (rAAA) vs. 15 non-ruptured AAAs (control group) were matched for pre-rupture imaging (first rAAA) and the initial post-rupture imaging (second rAAA) with two images in the asymptomatic control group (first and second control). The matching criteria were as follows: aneurysm diameter, sex, and time period between imagings. The biomechanical properties were analyzed with the finite element method (A4clinicsRE, Vascops GmbH, Graz, Austria). **Results**: Both groups had the same median aortic diameter of 5.5 cm in the first imaging but had significantly different aneurysm progressions with 6.9 cm (5.5–9.4 cm) in the second rAAA vs. 6.0 cm (5.1–7.3 cm) in the second control group (*p* = 0.006). The first rAAA, compared to the first control, showed significantly a higher peak wall stress (PWS) (211.8 kPa vs. 180.5 kPa, *p* = 0.029) and luminal diameter (43.5 mm vs. 35.3 mm; *p* = 0.016). The second rAAA, compared to the matched second control, showed a significantly higher PWS (281.9 kPa vs. 187.4 kPa, *p* = 0.002), luminal diameter (58.3 mm vs. 39.7 mm; *p* = 0.007), PWRR (0.78 vs. 0.49, *p* = 0.014) and RRED (79.8 vs. 56.5, *p* = 0.014). The rAAA group showed over-proportional averages, over the observation time, and an increase in PWS (nearly 10× faster in rAAA) and luminal diameter (nearly 4× faster in rAAA) per month. **Conclusions**: The finite element analysis of biomechanical properties could be used for the early prediction of an increased rupture risk in AAA patients. This was confirmed by matched imaging analyses before and after AAA rupture. Further multicenter data are needed to support these findings.

## 1. Introduction

The mortality in ruptured abdominal aortic aneurysm (AAA) remains high (±75%) considering both pre-hospital and in-hospital deaths, even though the mortality has somewhat declined over the years, from 86% to 74% [1,2]. The prediction of rupture for infrarenal aortic aneurysms is based on maximal aneurysm diameter as the first line decision making criterion for elective AAA repair, but it often fails to provide a patient-specific or individualized indication [3,4]. In the new 2024 ESVS guidelines, aneurysm size and growth speed remain the main criteria. A new recommendation is to continue surveillance in patients deemed unfit for surgery because of major comorbidities and short life expectancy [5]. It is important to identify patients with an elevated risk of rupture to offer early elective repair, but this should also include the patients that have a high perioperative risk and a low chance of rupture.

A biomechanical analysis towards estimating wall stress and strain can help in understanding aneurysm growth behavior and aneurysm rupture [6,7,8,9]. Aneurysm geometry, wall-related factors like wall thickness, intraluminal thrombus (ILT), tissue properties, blood flow properties and intraluminal pressure due to high blood pressure have been reported to influence aneurysm growth behavior [7,10,11,12].

The experimental methods for testing abdominal aortic aneurysm (AAA) tissues for the biomechanical analysis of aneurysm growth and rupture potentially involve integrating constitutive modeling and mechanical stress evaluation. One approach is the identification and application of finite strain constitutive models to characterize the nonlinear and anisotropic behavior of aortic wall tissues. This approach enables the simulation of tissue behavior under various loading conditions, providing insights into wall stress distributions [13,14].

Biomechanical properties have rarely been used to observe aneurysm growth behavior, especially in a predefined period of time. The focus could be placed on the direct comparison of the mechanical behavior of the aneurysm wall in ruptured and non-ruptured aneurysms.

In biomechanics, the finite element method (FEM) is a commonly used numeric method to simplify complex bodies, analyze mechanical problems and subdivide them into smaller parts called finite elements [15]. If it is used in terms of aortic diagnosis, the finite element method can be used to predict biomechanical parameters, such as stress in the aortic wall. Biomechanic parameters can be used to compare ruptured to non-ruptured aortic aneurysms and to analyze and interpret the growth behavior in both groups, an approach already proposed 20 years ago and further explored extensively [8,16].

Such an approach relies on the mechanical characterization of abdominal aortic aneurysm (AAA) tissues and their constitutive modeling towards FEM computation. Here, finite strain constitutive models capture the nonlinear mechanical properties of aortic wall tissues and allow the prediction of AAA wall stress under the complex multiaxial in vivo loading conditions [16,17,18].

The aim of this study is to identify geometrical/biomechanical parameters that can predict AAA rupture and identify abnormalities in the aortic wall mechanical loading that may be responsible for an elevated rupture risk.

## 2. Materials and Methods

### 2.1. Population and Study Design

Retrospective data from 1219 AAA patients (conservative, elective and acute repair), collected between January 2005 and April 2024, were screened for the inclusion in a pseudo-prospective single center study at the Department of Vascular Surgery, Medical University of Innsbruck, Austria. The inclusion criteria were as follows: infrarenal AAA with at least two measurements in two computed tomographic (CT) scans or one CT scan and one magnetic resonance (MR) scan. Pre-rupture and first imaging were CTs, and if no contrast enhanced spiral CT angiography (CTA) was available, contrast enhanced MRI was used instead. If there was more than one imaging from the same patient available, the CTA and the best time match was preferred. The second imaging was exclusively a CTA. The focus was laid on high resolution imaging with thin slices to identify structures exactly. The measurements were carried out axially aligned from two observers from the outer-to-outer aneurysm wall.

The patients of the ruptured AAA group (first rAAA + second rAAA) were analyzed, starting with pre-rupture imaging and the initial post-rupture imaging. Non-ruptured AAAs (control group) were time matched for the aneurysm diameter in their first imaging ±3 mm (first control), sex and time between the first and second imaging (in months), with the imaging in the control group conducted ±6 months at the point of rupture from the matching rAAA (second control).

The exclusion criteria were as follows: missing imaging or surveillance with ultrasound only, incomplete imaging, native imaging without contrast, dissected aneurysm, failed finite element analysis (no complete simulation possible due to technical reasons) or incomparable matching pairs that did not meet all of the required criteria.

The ethical approval for this study was obtained from the local ethics committee at the Medical University of Innsbruck (ECS 1301/2022).

### 2.2. Biomechanical Analyses

Patient-specific risk parameters were computed with A4clinicsRE (Vascops GmbH, Graz, Austria), a commercial biomechanical-based simulation software specially designed for analyzing AAAs. A4clinicsRE performs a finite element analysis and relates wall stress to a statistical estimate of wall strength, ultimately resting in a risk index. The biomechanical analysis is based on a geometrical model that represents the 3D anatomy of the infrarenal aorta and that is pressurized by the patient’s mean arterial pressure. Isotropic hyper-elastic constitutive models [19] describe the elastic properties of the aorta wall [17] and the ILT [20], and a predefined (but inhomogeneous) wall thickness is used. Overall, a large variability of the vessel wall [17] and ILT [21] elastic properties does not have a large implication on wall stress predictions. Our analyses were conducted from the infrarenal aortic segment to the aortic bifurcation, and the explicit boundary conditions, together with other modeling assumptions, are reported elsewhere [8,17,22].

Geometrical parameters, such as the maximum diameter, aorta volume and the volume of different types of tissues are derived from the individual AAA anatomy, and characteristic biomechanical parameters, such as peak wall stress (PWS), peak wall rupture risk (PWRR) and the rupture risk equivalent diameter (RRED) are extracted from the results of the finite element analysis. PWS reflects the highest wall stress, PWRR is the highest ratio between stress to strength, and RRED denotes the diameter at which the respective risk is seen, on average, in the AAA population. Further details concerning the applied method, verification and validation are reported elsewhere [18].

**Figure 1:**
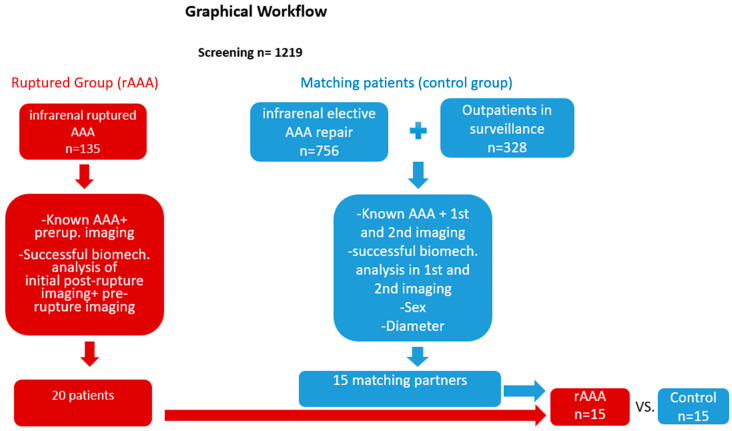
Graphical workflow on methodology of selection of patients suitable for analysis.

In summary, besides the CT-A images, the analysis required the patient’s age and blood pressure. If the blood pressure was available from the clinic’s patient-information-system, it was used; otherwise, a blood pressure of 140/80 mmHg was considered. In ruptured AAA patients in shock the blood pressure of 140/80 mmHg was also used. As the acute hypotension does not represent the assumed elevated long-time blood pressure, it would have distorted the results of the finite element analysis [23]. A graphical workflow is provided in Figure 1.

### 2.3. Data Processing and Statistical Analysis

The study data were collected and managed using REDCap 14.9.2 (Research Electronic Data Capture) electronic data capture tools hosted at the Medical University of Innsbruck. The best fitting patient regarding the first imaging diameter and months between the first and second imaging was determined. Sex was an absolute matching criterion, and age was the next pairing criterion at the same diameter and timeline.

All the descriptive data are given in medians (interquartile range (IQR)), and *p*-values below 0.050 were considered significant. Statistics, such as descriptive analyses for age, aortic diameter, sex, nicotine and diabetes, were conducted with IBM SPSS Statistics. Further analyses for PWS, PWRR, luminal diameter and RRED were tested for normal distribution and then analyzed with the Mann–Whitney U Test. The averaged growth rates were calculated by comparing the diameter values of the first rAAA and the second rAAA, respectively, with the first control and the second control, and dividing the difference by the observation time in months. The averaged increases in PWS and luminal diameter were calculated accordingly.

## 3. Results

### Patient Characteristics

From a total of 1219 patients screened for the diameter of their aortic aneurysms, 20 patients with ruptured aortic aneurysms and available pre-imagings were identified (rAAA). A total of 5 patients did not have a time match and were excluded from further analyses. Patients were time matched to identify 15 (rAAA: n = 15) fitting patients with the same aortic aneurysm diameter in the first imaging, sex and duration of follow-up (control group: n = 15). In total, 30 patients (15 matched pairs) of ruptured vs. non-ruptured AAA patients could be identified with matching criteria and successful biomechanical analyses, and they were pseudo-observed for an individual time for each pair, meaning the time until the rupture happened in the rAAA group. The control group contained eight elective repairs (four OAR and four EVAR’s) and seven conservatively treated patients. The reasons for conservative treatment were that patients were unfit for surgery or had limited life expectancy (n = 3), missed controls (n = 2) and refused intervention (n = 2).

The rAAA group had a median age of 81 years (IQR 73–84 years) in the pre-rupture imaging group (first rAAA), compared to 75 years (IQR 67–81 years, *p* = 0.12) in the control group (first control), showing that the control group was of similar age as the rAAA group at the initial imaging (Table 1).

At the time of rupture (second rAAA) vs. second control group imaging (second control), patients had a median age of 82 years (IQR 76–86 years) vs. 76 years (IQR 69–82 years, *p* = 0.070).

As shown in Table 1, patients in the rAAA and control groups were chosen based on the same baseline median aortic diameter (both groups 5.5 cm (5.1–5.9 cm [first rAAA] vs. 5.1–5.7 cm [first control]). At the second imaging the difference in diameter was significant at 6.9 cm vs. 6.0 cm (6.3–7.2 cm vs. 5.8–6.2 cm, *p* = 0.006). Patient characteristics, such as smoking status and diabetes were comparable, as depicted in Table 1.

The median follow-up duration was 13 months (7–29 months) in the rAAA and 15 months (11–30 months) in the control group.

As depicted in Table 2, the comparison of the first rAAA analysis and first control analysis showed a significantly higher PWS (211.8 kPa (191.3–256.7 kPa) vs. 180.5 kPa (158.9–206.7 kPa), *p* = 0.029) and luminal diameter (43.5 mm (38.7–51.2 mm) vs. 35.3 mm (30.4–44.3 mm), *p* = 0.016) prior to aortic rupture. There was a clear tendency for higher PWRR and RRED in the first rAAA, compared to the first control.

The second rAAA analysis, compared to the second control analysis, showed significantly higher PWS (281.9 kPa (259.5–324.4 kPa) vs. 187.4 kPa (175.6–253.3 kPa), *p* = 0.002), luminal diameter (58.3 mm (49.2–68.9 mm) vs. 39.7 mm (31.1–46.2 mm), *p* = 0.007), PWRR (0.78 (0.67–0.99) vs. 0.49 (0.37–0.81), *p* = 0.014) and RRED (79.8 mm (71.4–94.8 mm) vs. 56.5 mm (45.1–82.2 mm), *p* = 0.014).

**Figure 2:**
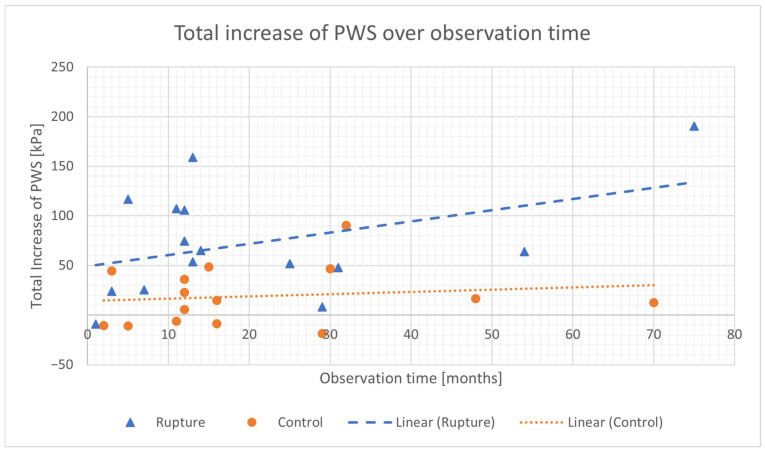
Increase in PWS (peak wall stress) as a function of observation time in comparison between ruptured (rupture) and asymptomatic (control group) infrarenal aortic aneurysm cases.

**Figure 3:**
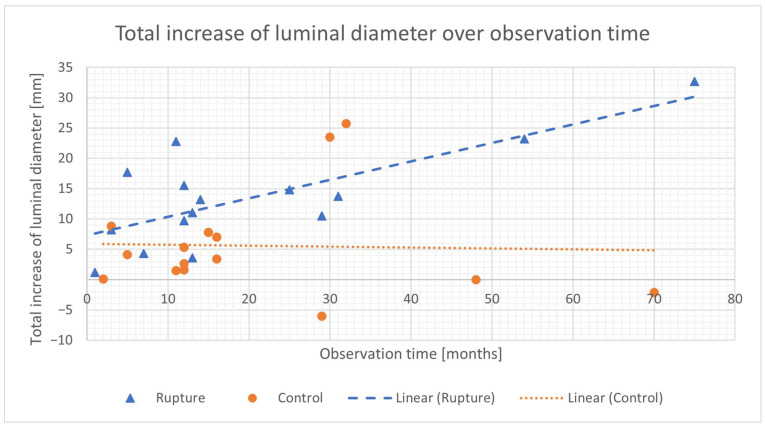
Increase in luminal diameter as a function of observation time in comparison between ruptured (rupture) and asymptomatic (control group) infrarenal aortic aneurysm cases.

The averaged, over the observation time, diameter growth rate was significantly higher in the rAAA group, compared to the control group (1.0 mm/month (0.47–1.23 mm/month) vs. 0.33 mm/month (0.18–0.67 mm/month), *p* = 0.005). The averaged increase in PWS (4.12 (1.54–8.81) vs. 0.47 (−0.55–+2.82) kPa/month, *p* = 0.012) and luminal diameter (0.81 (0.44–1.29) vs. 0.22 (0.05–0.78) mm/month, *p* = 0.008) were also significantly higher in the rAAA group, as visualized in Figure 2 and Figure 3 as a function of observation time in the comparison between ruptured and non-ruptured infrarenal aortic aneurysm cases. ILT thickness values did not differ between the two groups. (first rAAA compared to first control *p* = 0.90, second rAAA compared to second *p* = 0.56; *p* = n.s.).

In the ruptured group, the total increase in the luminal diameter over the observation time correlated significantly with the observation time (r = 0.748 *p* = 0.001, 90% CI, 2.64 to 27.00 mm). The total increase in PWS over the observation time correlated with the observation time, although not significantly (r = 0.417 *p* = 0.12, 90% CI, 1.12 to 171.38 kPa). In the control group, the total increase in the luminal diameter and the total increase in the PWS correlated with the observation time, although not significantly (luminal diameter: r = −0.081 *p* = 0.78, 90% CI, −3.66 to 24.38; PWS: r = 0.265 *p* = 0.34, 90% CI, −14.02 to 65.10 kPa).

## 4. Discussion

**Figure 4:**
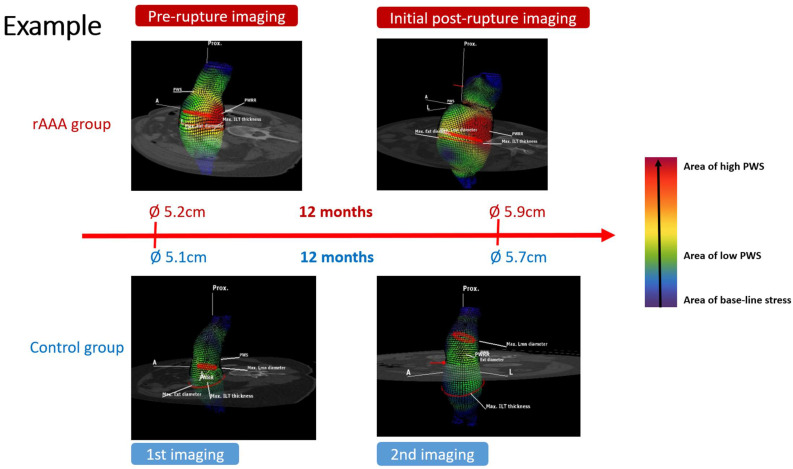
Example of biomechanical output from one matched pair. Areas of high stress are colored in red and areas of lower stress are colored in yellow to green. Blue implies base-line stress.

This study proposes a potential method for the prediction of rupture risk (Figure 4) and to improve our ability to perform individualized decision making in AAA repair.

The population was matched for the timing of imaging, diameter, sex and age to find pairs and reduce co-factors to accentuate the differences in biomechanic characteristics. Since PWS was found to increase with diameter, it was important to exclude diameter-related biases [24]. Therefore, the diameter was matched in the first imaging of both groups.

PWS and luminal diameter were increased even in the pre-rupture imaging (first rAAA), months before the rupture. An insignificant trend for increased parameters in this group was also shown in PWRR and RRED, probably due to the small sample size. It was expected that the biomechanic parameters would be higher in rAAAs, compared to the matching group, since recent research correlated the elevation of these parameters with rupture [25,26].

In the clinical approach of “watchful waiting” during the observation of AAAs, FEM could improve the understanding of structural changes in the aortic wall by monitoring the stress and deformation of the aortic wall over time. Therefore, a patient-specific simulation model could be used to optimize decision making towards uncovering the individual elective aneurysm repair threshold. The process is semiautomatic, so that clinicians can learn and use the analysis program in a reasonable amount of time. Due to the small sample sizes in previous studies, the use of biomechanical parameters for clinical decision making lacks evidence to be included in the guidelines [27,28].

Another study focused on basic demographics and artificial intelligence-based rupture risks. Sex, smoking, body size and aneurysm diameter influence the biomechanical rupture risk, according to Skovbo et al. [29]. In our study, basic demographics like sex and smoking were also considered in the analysis. Smoking, however, did not differ between the two groups in our cohort.

The use of artificial intelligence is becoming increasingly important in assessing the risk of aortic aneurysm rupture and supporting individualized decision making [29].

All patients had imaging performed with a known aneurysm in their history. The reasons why an elective repair was not conducted were different in our series. Patients were either not fit for surgery, did not have the suitable anatomy for endovascular repair or preferred a conservative treatment; other patients were lost in follow-ups. Interestingly, some of the matched non-ruptured aneurysms reached a diameter between 8 and 10 cm without any sign of rupture or symptoms. Thus, the question is what are the determining factors that cause some aneurysms to rupture with small diameters, while others reach diameters of 8–10 cm, remaining intact. Aneurysm diameter does not seem to be the only important factor. Shape also seems to have an impact on stress on the aortic wall. Nathan et al. showed that the normalized wall stress (peak wall stress dependent on maximal aneurysm radius) of saccular aneurysms of the descending thoracic aorta was greater than in fusiform ones [30].

The importance of biomechanical analyses in the prediction of rupture in AAAs was shown in previous studies and was summarized in systematic reviews and meta-analyses [28,31]. The fidelity of biomechanical simulations is continuously increasing and, to date, no strong consensus on which particular modeling assumptions should be used have been emerged. Besides model development, more clinical validation [8]) is one of the most pressing needs to progress in the field. In a study by Erhart et al., areas with a high rupture risk in FEM analyses were correlated with histological wall disintegrity in samples taken during elective aortic repair. Areas with the highest rupture risk index showed the increased histopathological degeneration of the aortic wall [32]. These results align with the depicted tendency of a high PWRR in the first rAAA group, and the significantly higher PWRR in the second rAAA analysis at the time of rupture, compared to the control group. However, contradictive results, in terms of predicted rupture locations, have also been reported [33].

The majority of studies focus on the differences between ruptured and non-ruptured aortic aneurysms at the time of rupture [24,28]. Biomechanical differences in these groups have proven to be significant, but rarely addressed predicting rupture risk. There is scarce data focused on the time before the rupture occurs. There is a recent study about the biomechanical analysis of serial CT-angiographies (CTa) which focused on the identification of factors associated with aortic rupture risk in a survival model. Aneurysm volume, PWS and the peak wall rupture index were significantly associated with rupture risk, which supports our findings. The results were then adjusted for sex with similar results. Surprisingly, in a multivariate analysis adjusted for size and sex, PWS was not significantly associated with the risk of rupture, which possibly can be explained, that not a single parameter but the combination of two or more parameters can predict the rupture more accurately [34].

A possible challenge for the clinical application of biomechanical analyses with specialized software and 3D models is the learning curve. Training is needed for successful and reliable data analysis. But, once established, the reproducibility of patient specific 3D FEM models from biomechanical information is high [35,36]. A high resolution of the imaging modality is important to allow for an accurate geometry reconstruction and to decrease model based variability. In CTA, information about the vessel wall, compared to intraluminal thrombus, is sometimes difficult to differentiate. One study reports that peak wall stress differs between constant wall thickness models and variable wall thickness from CT models [37]. AAA wall thickness is heterogeneous (1.58–2.00 mm) and AAA wall strength is inverse correlated to wall thickness [8,18]. As a thick layer of thrombus thins the wall underneath it, this effect was incorporated in our biomechanical models; the wall thickness varied between 1.5 mm (not covered by ILT) and 1.33 mm (covered by a thick ILT layer) [8,38]. Further studies for the possible impact of wall texture and thickness could also improve the accuracy of finite element analysis [39].

## 5. Limitations

The small sample size was a limitation of our study. To find suitable matching partners, more than a thousand cases were screened. According to current and previous guidelines, the indication for aortic repair was mostly at 5.0 cm (women) and 5.5 cm (men) [5]. Some of the ruptured cases had their pre-imaging at a diameter of more than 5.5 cm, so there was the possibility that their possible non-ruptured matching partner already underwent surgery or endovascular repair after the first imaging and did not reach the required observation time or did not have another imaging to be matched. Some patients of the control group did not have a matchable CTA imaging, and so MR scans had to be taken. Both CT or MR scans may be used to segment the aneurysm and provide the geometry for finite element analyses. While a larger slice thickness (>2 mm) complicates the segmentation and requires more user interactions, it does not have a significant influence on the results of biomechanical analyses. The A4clinicsRE software uses active segmentation models, a method that is independent from the resolution of the underlying images [40].

## 6. Conclusions

This study offers a method for the prediction of rupture risk and to improve our ability to allow for individualized decision making in AAA repair. An elevated peak wall stress and luminal diameter were early predictors for AAA rupture even months before the aortic event. Significant differences in these two parameters, calculated as growth rates per month, could provide even more important information on AAA growth behavior and rupture risks for AAA patients. This method could help to identify high risk patients with elevated biomechanical parameters and over-proportional increases in PWS and luminal diameter between CT controls to undergo early elective surgery, and also identify low risk patients for aortic rupture with stable biomechanical parameters, who would probably not benefit from early elective repair and would rather have suffered from perioperative complications or die from any other cause other than their aneurysm in their remaining lifetime. This new and innovative prediction tool warrants further studies with larger sample sizes and possible implications for future treatment decisions.

## Figures and Tables

**Table 1:** Baseline characteristics of ruptured AAA group and control group. Continuous data are presented as median + interquartile range and were tested with the Mann–Whitney U Test. Nominal data are presented as numbers (%). *p*-values below 0.050 were considered significant.

	Ruptured Group (n = 15)	Control Group (n = 15)	*p* Value
Male Sex	13 (87)	13 (87)	
Female Sex	2 (13)	2 (13)	
Age at First CT	81 (73–84)	75 (67–81)	0.12
Age at Second CT	82 (76–86)	76 (69–82)	0.070
Diabetes	5 (33)	4 (27)	0.70
Smoking Status			0.69
Non-Smoker	5 (33)	6 (40)	
Current Smoker	7 (47)	3 (20)	
Former Smoker	3 (20)	6 (40)	

**Table 2:** Biomechanical analysis: continuous data are presented as median + IQR and were tested with the Mann–Whitney U Test. *p*-values below 0.050 were considered significant. PWS = peak wall stress; PWRR = peak wall rupture risk; and RRED = rupture risk equivalent diameter.

	Median		*p*-Value	Median		*p*-Value
	First rAAA	First Control		Second rAAA	Second Control	
Aortic diameter [mm]	55 (51–59)	55 (51–57)	0.57	69 (63–72)	60 (58–62)	0.006
PWS [kPA]	211.8 (191.3–256.7)	180.5 (158.9–206.7)	0.029	281.9 (259.5–324.4)	187.4 (175.6–253.3)	0.002
PWRR [-]	0.52 (0.44–0.62)	0.43 (0.37–0.56)	0.059	0.78 (0.67–0.99)	0.49 (0.37–0.81)	0.014
Luminal diameter [mm]	43.5 (38.7–51.2)	35.3 (30.4–44.3)	0.016	58.3 (49.2–68.9)	39.7 (31.1–46.2)	0.007
RRED [mm]	58.9 (52.0–67.2)	50.6 (45.3–62.5)	0.065	79.8 (71.4–94.8)	56.5 (45.1–82.2)	0.014
ILT Thickness [mm]	19.6 (10.5–23.9)	21.5 (14.6–24.4)	0.90	20.4 (14.4–29.9)	24.0 (18.7–26.9)	0.56

## Data Availability

The data that support the findings of this study are available upon request from the corresponding author, [KG]. The data are not publicly available due to ethical restrictions.
